# Minority Influence and Degrowth-Oriented Pro-environmental Conflict: When Emotions Betray Our Attachment to the Social Dominant Paradigm

**DOI:** 10.3389/fpsyg.2022.899933

**Published:** 2022-06-29

**Authors:** Robert A. T. Avery, Fabrizio Butera

**Affiliations:** Institute of Psychology, University of Lausanne, Lausanne, Switzerland

**Keywords:** minority influence, emotions, degrowth, change, conflict, pro-environmental action

## Abstract

If today the anthropogenic origin of climate change gathers almost total scientific consensus, human pro-environmental action is not changing with sufficient impact to keep global warming within the 1.5° limit. Environmental psychology has traditionally focused on the underlying barriers towards more pro-environmental behaviours. Emotions—like fear or anger—may act as such barriers especially in case of radical change (e.g., degrowth). While minority influence has been extensively applied to understand societal change, it has rarely been applied to understand the emotional responses that may hinder counter-normative pro-environmental messages. However, past literature on emotions shows that, in challenging situations—the likes of radical minority conflict—people will tend to use their emotional reaction to maintain societal status quo. Two studies investigated how participants emotionally react towards a counter-normative pro-environmental minority message (advocating degrowth). A qualitative (thematic analyses) and a quantitative (emotional self-report paradigm) studies showed that participants report emotions that allow them to realign themselves with the cultural backdrop of the social dominant paradigm (growth), thus resisting change. Specifically, although all participants tend to demonstrate higher proportions of control-oriented emotions, men do so more. These effects, as well as questions of cultural and ideological dominance, are discussed considering barriers towards pro-environmentalism.

## Introduction

« I hold a vision of this blue green planet, safe and in balance. At the end of the Fossil Fuel Era, we are emerging to a new reality. We are ready to make the next leap - as momentous as abolishing slavery or giving women the vote. » *–* Elizabeth May

Elizabeth May declared in her quote that *we are ready to make the next leap*; but are we? It would, indeed, seem that through years of challenge, environmental activism has, step by step, managed to positively sway western public opinion about environmentalism (Rapley et al., 2014; Taylor, 2019; Bevan et al., 2020). Pro-environmentalism is becoming increasingly normative, and we may now be under the impression that governments, companies, and population are all on the same page. Given this evolution, the current context warrants new research that tests people’s reactions towards pro-environmental active minority sources that are able to produce conflict, the crucial ingredient of minority influence and social change (Moscovici, 1976). We, thus, decided to study the effect of minorities whose pro-environmental influence is aimed at more fundamental changes, such as degrowth. Specifically, the present research studies how people tend to respond defensively towards such policies because they radically threaten the growth tenet of the current western social dominant paradigm. Furthermore, we will argue that men, more than women, are particularly concerned with such threat.

### Minority Influence and the Apparently Consensual Nature of Pro-environmentalism

Current pro-environmental activism and minority influence is apparently rendered unnecessary by the consensual nature of pro-environmentalism. Judging by the Intergovernmental Panel on Climate Change’s report (IPCC, 2022) the current pro-environmental actions undertaken by people, institutions and governments are not sufficient to steer human activity to remain within planetary and ecological boundaries—e.g., in terms of climate change, nitrogen and phosphorus loading, biodiversity, land conversion, etc.—, while at the same time respecting social foundations such as health, education, gender equality, peace and justice, etc. (Raworth, 2017). The problem is that pro-environmentalism is no longer the controversial topic it once was, as indicated, for instance, by the high levels of acceptance for political policies aiming at environmental protection (European Commission, Directorate-General for Communication, 2019). Public opinion is evolving, and environmental values are becoming part of mainstream overt values. For example, in current western cultures, it is no longer acceptable, for individuals, countries, and firms alike, to openly display disregard for the environment (Félonneau and Becker, 2008; Chelli et al., 2018; Falkner, 2021). Moreover, scientific consensus about the anthropogenic origin of climate change is close to unanimity, with higher levels of consensus among experts of climate change (Cook et al., 2016; Lynas et al., 2021). As the current cultural paradigm^1^ (Pirages and Ehrlich, 1974) includes more and more normative positive attitudes towards the environment, it becomes increasingly hard for environmental activism to elaborate a societal conflict and, thus, provoke change in actual behaviours (Forno and Wahlen, 2022).

### Minority Conflict and the Challenge of Consensus

Over 50 years of research on minority influence have shown that activists, minority movements and counter-normative groups may achieve social change, to the extent that they can induce conflict in their targets (Moscovici, 1976; Simon and Klandermans, 2001; Butera et al., 2017; Papastamou et al., 2017). Moscovici’s seminal work and his conversion theory have drawn a great deal of attention on the mechanisms that groups lacking social power can use to disrupt consensus and initiate social change: Through the conflict introduced by their counter-normative claims, and to the extent that they consistently maintain these claims over time, active minorities can lead their targets—the population—to consider and validate the content of the newly proposed norms, and step by step change their attitudes and eventually their behaviours (e.g., Moscovici, 1980).

Some research has shown that minorities are particularly effective when their claims are congruent with the societal Zeitgeist (Pérez et al., 1994); however, this does not prevent minorities to negotiate in a flexible way with the population, while maintaining a hard-line, disruptive position in their confrontation with institutional sources of power (e.g., Mugny, 1982; Shuman et al., 2021). What happens, then, when activist groups insist in promoting norms with which the whole society apparently agrees? They run the risk to be considered as extremists, utopians, or fools (Papastamou, 1986), or simply be unable to bring targets to elaborate conflict (Pérez and Mugny, 1996). Accordingly, several pro-environmental groups have resolved to more and more antagonistic strategies to elicit a conflict.

### Degrowth as a Conflictual Minority Position

Many recent examples show that conflict can be elicited even in societies where protection of the environment has become a pervasive norm: (a) Environmental activism has been embodied by children outright blaming the political elite for ignoring the scientific facts pointing towards an ecological crisis future (Zulianello and Ceccobelli, 2020); (b) Extinction Rebellion, one of the largest multinational environmental activism organisations holds the alarmist word ‘extinction’ in its very title (XR; Extinction Rebellion (XR), 2020); (c) Youth climate change activists employ disruptive forms of dissent (e.g., corporate property occupation by youth in Norway) to manifest for climate (O’Brien et al., 2018); and (d) Work on strategic narratives towards climate change have identified five discourses in the United Kingdom media following the 2019 IPCC report, all of which are related to either *limited time left (or emergency)*, *collapse*, *destruction* or *damage to our planet* (Monroe et al., 2019). However, as the apocalyptic framing of climate change has become part of the mainstream media discourse, fear appeals may desensitise individuals and become less effective in generating conflict (Cassegård and Thörn, 2018). This has led some environmental activists to challenge certain foundations of the current hegemonic western worldview defined as the Dominant Social Paradigm (DSP; Dunlap and Van Liere, 1984) in favour of a New Environmental Paradigm (NEP; Dunlap, 2008).

Challenging the growth tenet of the DSP holds a great potential for elaborating conflict. On one hand, belief in growth is such a strong ideology that most of the current environmental actions and policies are oriented around the idea of green growth (Kilbourne et al., 1997; Urhammer and Røpke, 2013; Urhammer, 2015); a rather interesting example of this phenomenon is described in Gifford’s “dragons of inaction” under the name technosalvation (the belief that humankind will develop sufficient technological solutions to our environmental problems, O’Riordan, 1995; Kilbourne et al., 2002; Gifford, 2011). On the other hand, Degrowth Oriented Pro-Environmental (DOPE) policies can be defined as solutions based on anti-growth economic models; they include projects of voluntary individual, industrial, and societal shrinking of production and consumption aimed at social and ecological sustainability that are congruent with planetary boundaries (Rockström et al., 2009; Demaria et al., 2013; Kallis et al., 2018). DOPE policies are, therefore, conflictual in the sense that they are contrary to the growth tenet of the current DSP.

Policies that challenge the DSP are threatening to the general population. First, participants have been shown to react towards DOPE policies with more negative emotions simply because the use of ‘degrowth’ in the title created feelings of reactance (Drews and Reese, 2018). Second, accepting DOPE policies implies redefining human relationship to nature (e.g., humans are part of nature) which, in turn, reminds us of our own natural boundary—death. This reminder of our own mortality could lead to understanding DOPE policies as an existential threat (Koller, 2020). Third, degrowth is also used as a political tool to convey the desire for radical change (both social and economic). In regards to social change, a more egalitarian system could be considered a threat to those benefiting from their dominant position; and in regards to economic change, those who do not currently benefit from a wealthy and privileged situation may perceive it as an even greater pressure on their livelihood (Akbulut et al., 2019; Muradian, 2019; Stanley et al., 2021). Fourth, accepting DOPE policies often implies handing over the control of certain behaviours to an external source of control (e.g., governments control what type of car one can drive), and decrease in control has long been shown to be considered a threat in itself (Brehm, 1966; Whalen, 1998). Given the threatening potential of DOPE policies, some pro-environmental minorities currently advocate DOPE policies to actively challenge society’s dominant paradigm and elicit conflict; but how will people react to such policies?

### Responding to DOPE Policies Proposed by Minority Influence: The Importance of Emotional Responses

The discourse around pro-environmentalism is often emotionally ladened. On one side, the emotional framing of climate-related policies (hope, fear, and worry) help explain up to 50% of variance in people’s support (Smith and Leiserowitz, 2014) and, thus, active minorities often rely on affect to generate the perception of climate emergency and promote mitigation action (Doulton and Brown, 2009). On the other side, shared emotions such as hope and anger are vital in determining intention to participate in collective action (Rees and Bamberg, 2014; Fritsche and Masson, 2021). Although the field of environmental affect still needs integration (Pihkala, 2022), it seems important to understand the role of emotions in the context of pro-environmental minority influence; especially when considering that DOPE policies could be considered a threat, as noted above.

Thus, in the case of DOPE policies proposed by an active minority, the response is likely to be defined by an emotional response aimed at the control of threat (Bolderdijk and Jans, 2021). Research on minority influence has not focused on emotions in a systematic manner. Some studies have shown that minority influence does elicit emotional arousal (Nemeth and Wachtler, 1983; see also Maass and Clark, 1984) and that in some cases minority positions are met with promotion-related emotions, such as cheerfulness (Falomir-Pichastor et al., 2008), but more needs to be done to integrate research on emotional responses with research on minority influence. The present research wishes to contribute to such an endeavour.

Emotional responses to threat are control oriented, as revealed by the major control-oriented modal emotions described in the Geneva Emotion Wheel (GEW; a tool for measuring self-reports of emotions; Scherer, 2005). The GEW is a visual representation of 20 discrete emotions, organised on two dimensions, valence and control orientation, on the basis of Scherer’s appraisal process model of emotions (Scherer, 2001). This model explains that emotions are more highly elicited and differentiated when their appraisal is driven by either goal conduciveness (i.e., valence) or coping potential (i.e., control orientation). Based on the appraisal process model of emotions, we can hypothesise that when members of a group report anger, contempt, and disgust as a threat response they are, in fact, responding to the threat with emotions high in coping potential, or in other words, control-oriented emotions. However, different groups should appraise DOPE policies as more or less of a threat depending on their roles and status, and therefore need for control.

### Emotional Responses to Threat: A Gendered Control-Oriented Response

People’s emotional reactions depend on their gender role. Research on gender roles and emotions indicates that men and women express and regulate their emotional expressions to fulfil what is expected of them (Fischer, 1993; Fischer and Manstead, 2000, 2016; Brody et al., 2016). Namely, on the one hand, girls and women are taught and expected to suppress anger and frustration, as well as be quiet and accommodating, whereas on the other hand, boys and men are taught and expected to suppress anxiety, fear, and sadness as well as express anger (Schwalbe, 2015; Gansen and Martin, 2018). Referring to the aforementioned appraisal model of emotions (Scherer, 2001), we could argue that, generally, women are socialised to respond with low control-oriented emotions and men are socialised to respond with high control-oriented emotions.

People’s emotional reactions reflect their status which, in turn, is linked to their gender within the dominant social paradigm. Social dominance theory (SDT) posits that the higher one’s group’s status, the higher their social dominance orientation (SDO; Sidanius and Pratto, 1999). Furthermore, SDT predicts that, all else equal, men will systematically score higher in SDO than women because of (but not exclusively due to) their dominant status within said society (Sidanius et al., 1994). Although the invariance of gender has often been debated, recent research on the effect of gender socialisation on SDO reveals that self-stereotypes and gender identities explain significant amounts of variance in SDO (Dambrun et al., 2004; Foels and Pappas, 2004; Schmitt and Wirth, 2009; Snellman et al., 2009). In other words, men are particularly attached to their status, and therefore motivated to maintain it. Transposed to the issue at hand, this implies that men would imply that men are particularly motivated to maintain the dominant social paradigm that constitutes the societies which afford them their dominant status. Given that men are particularly attached to their status and that they are socialised to express and identify emotions that are coherent with high coping potential, we hypothesise that men should be more threatened that women by DOPE policies and, hence, express even more high control-oriented emotions.^2^

### The Present Research

The present article reports two studies aimed at exploring the conjecture whereby the threat resulting from degrowth oriented pro-environmental (DOPE) policies proposed by an active pro-environmental minority would elicit control-oriented emotional responses, more strongly so for men. Study 1 is a qualitative exploration of people’s spontaneous verbal responses when asked what emotions different DOPE policies provoked. Study 2 formally tests the hypothesis derived from the findings of study 1. We recorded participants emotional reactions towards the same DOPE policies as in study 1 and compared the part to whole ratio of high control-oriented emotions they selected. In study 2, we expected that the part to whole ratio of high control-oriented emotions would be greater than .5, revealing a tendency to express these emotions in case of threat, and that the ratio would be greater for men than for women.

It important to note at this point that with the aforementioned variables we are not studying social change *per se*. Actually, very little research in the area of minority influence has used variables that directly pertain to social change, as recently noted by Prislin (in Press). However, as emotions have been shown to play a role in social change (Jasper, 2011), the aim of this study was to research antecedents of such change focusing on the emotions provoked by minority influence regarding environmental issues.

## Study 1

The aim of study 1 was to analyse, in a diverse sample of participants, the verbal self-reported account of their feelings when faced with (DOPE) policies. Specifically, study 1 was interested in highlighting the social function of emotions as control-oriented responses to threat.

### Method

#### Research Design Overview

A series of semi-structured interviews were conducted by two independent researchers. Interview transcripts were coded in light of work on thematic analysis (Clarke and Braun, 2017), allowing emotion responses to naturally emerge from open questions. The main advantage of thematic analysis is that it allows to combine insights from both pre-existing theory (Scherer’s appraisal model of emotions in our case; Scherer, 2005) and bottom-up data observations that would not strictly pre-existing theory. Furthermore, the coding analysis was conducted following the consensual qualitative research method (CQR; Masdonati et al., 2017). Linking this approach with thematic analysis allows to account for the multiplicity of viewpoints on a given reality and helps researchers reveal systematic processes through the iterative process of a relatively small corpus of interviews. The process of systematically comparing and challenging each other’s coding and themes allows for a particularly transparent and reliable method of analysis.

#### Study Participants: Recruitment and Description

Participants were invited for an individual interview at the request of either researcher. To ensure minimal interviewer-interviewee contact and familiarity, the two researchers invited potential participants among their acquaintances and then exchanged the contact details to conduct the interviews. We sought out participants depending on a pre-defined grid of target audience. To recruit a diverse sample, the grid crossed four variables: gender (male, female) × place of activity (urban, rural) × age (based on employment stages of life: 18–26, 27-retirement, retired) × highest level of education obtained (higher education or not). We intended to recruit a sample of 24 participants. However, due to the COVID-19 pandemic, only 21 participants were finally recruited.

The final sample for the interviews was composed of a wide variety of profiles from young urban students to elderly people who grew up and lived in the countryside (aged from 20 to 76 years old). A summary of specific number of cases (*N*s) by demographic attributes can be found in Table 1.

**Table 1:** Demographic information for participants.

Demographic characteristic	By age-employment status		
	Student (18–26) (*n* = 9)	Employed (27—retired) (*n* = 6)	Retired (retired -) (*n* = 6)
Gender			
Male	4	4	2
Female	4	4	3
Place of activity (life)			
Urban	3	3	0
Rural	5	5	5
Highest Education			
No higher education	4	4	4
Higher education	4	4	1
Interviewed during COVID19 pandemic			
Yes	2	2	2
No	6	6	3

#### Data Collection Procedure

Both researchers followed an interview script (see Supplementary Material A for the original French version). First, participants were greeted, and the recording started on the interviewers’ smartphone (for the videoconferences, the audio was extracted from the recorded video). Participants were asked to sign (or to orally accept, for the virtual variant) an ethics form on which it was specified that they would be recorded. Second, participants were asked to read what they believed to be an extract of a manifesto. The manifesto was written in the prototypical rhetoric of a radical pro-environmental minority and contained three examples of DOPE policies [see section about the manifesto below and full version (French) in Supplementary Material B]. This was included to ensure all participants were exposed to the same level of conflict. Then, the interviewers reminded them that the answers were anonymous and that they were free to imagine other examples of pro-environmental policies than the ones that would be read out. Once the participant’s consent was received and following the interview script, researchers introduced the first DOPE policy. For each DOPE policy, participants were asked what emotions the policy elicited. This process was repeated three times (for each policy). Finally, participants were asked some demographic questions and whether they had any other comments or questions before terminating the recording by debriefing and thanking participants for their time.

#### Degrowth-Oriented Pro-environmental Manifesto

The bogus manifesto extract was written by the experimenter as if it came from a group of environmental activists named ‘Alarm Swarm.’ The three main arguments were derived from meta-issues of Crist (2018) as well as real (albeit hypothetical) examples of policies to counter these issues. These three topics dealt with: (1) (over)-population, as in the argument that the world cannot sustain such a high population’s consumption and pollution; (2) Over-consumption of natural resources, such as in the clothing industry; and (3) infrastructural incursions into nature, such as the industrial meat consumption industry that destroys forests to plant food for the cattle (Porter, 2014; Crist, 2018; Affolter and Junod, 2019; Louis and Martin, 2019). The rationale for using these meta-issues was that they are coherent with the most efficient individual pro-environmental behaviours described by Wynes and Nicholas (2017) and therefore, theoretically consistent with what degrowth-oriented pro-environmental active minority influence could formulate (Supplementary Material B).

#### Issues Related to the Perception of the Manifesto

We make the case that the influence source of the manifesto is a minority, which is plausible because degrowth is by far a minority position and Alarm Swarm is portrayed as a radical activist group. Furthermore, we have presented here literature and study findings making the point that DOPE policies can be perceived as threatening; these considerations remain, however, conjectures.

To substantiate these conjectures, we conducted a manipulation check.^3^ While one half of the British participants read the bogus manifesto as presented in the present article, the other half, read another manifesto from Alert Swarm which was in all points similar except for the policies that were this time growth oriented (GOPE). After having read one or the other manifesto, all participants had to rate (a) the descriptive normativity of the manifesto (“On a scale from 0 to 100%, please indicate, in your opinion, the proportion of the United Kingdom population that would agree to the kind of policies/solutions proposed by the activist group.”), (b) the prescriptive normativity (“On a scale from 1 (total disruption) to 7 (total conformity), please rate the degree to which the solutions proposed by Alert Swarm are compatible with how the UK functions”) and finally (c), on a scale from 1 to 7 how threatening the policies were perceived.

Participants found the DOPE manifesto (*M* = 34.88, *SD =* 20.63) less descriptively normative than the GOPE manifesto (*M* = 40.44, *SD =* 20.37), *t*(528) = −3.11, *p* < 0.01, Cohen’s *d* = 0.27. Participants also rated the DOPE manifesto less prescriptively normative (*M* = 3.17, *SD =* 1.23) than the GOPE manifesto (*M* = 3.48, *SD =* 1.34), *t*(523) = −2.7, *p* < 0.01, Cohen’s *d* = 0.24. Thus, both manifestos attributed to an activist group were perceived as presenting a descriptively and prescriptively minority norm; but more so when that norm advocated degrowth rather than growth. Moreover, in line with our conjectures, participants rated the DOPE policies as more threatening (*M* = 4.04, *SD =* 1.26) than the GOPE manifesto (*M* = 3.39, *SD =* 1.07), *t*(556) = 6.44, *p* < 0.001, Cohen’s *d* = 0.55.

#### Data Analysis

All interviews were conducted, recorded, transcribed, and coded by the first author and a student research assistant. All transcriptions were then imported in to RQDA (Qualitative Data Research package for R; Ronggui, 2016). Analysis followed a systematic coding strategy informed by CQR (Masdonati et al., 2017) and elements of thematic analysis (Braun and Clarke, 2006). The data analysis protocol combined thematic analysis and CQR, yielding six main steps (data familiarisation, code generation, search for themes, theme review, theme definition and naming, and report write-up). All steps were conducted in conjunction with the research team (the two authors and a student research assistant, as mentioned in Supplementary Material). For a detailed account of the work accomplished for each step, see Supplementary Table S1.

### Results

The results are organised in two sections. First, we give an overview of the codes used to categorise participants’ emotional responses, focusing on two generally prevalent codes, namely avoidance and sadness. Second, we present the gendered aspect of emotional accounts, focusing on avoidance, fear and anger and their control orientation.

#### General Characteristics of Emotional Responses

**Figure 1:**
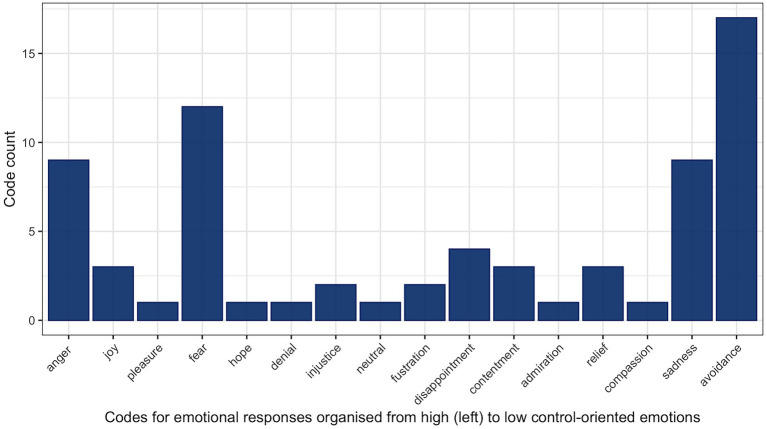
Count for each emotion code.

Figure 1 provides an overview of all codes used to describe the emotions identified by participants or by the research team’s interpretations; whenever possible, emotions were grouped under codes coherent with the emotions labelled in the GEW (appraisal model of emotions; Scherer, 2005; Scherer et al., 2013a). The list of emotions in Figure 1 is organised on the *x*-axis from high to low control-oriented emotions. In addition to the discrete emotions found on the GEW, we included: hope, denial, injustice, neutral in the middle of the continuum and, at the end of the scale, the code ‘avoidance’ describes the occurrences where participants did not identify any emotions in their response. Although the overall identification of positive and negative emotions was roughly equal, positive emotions (joy, pleasure, hope, contentment, admiration, relief, and compassion) had relatively low prevalence compared to their negative counterparts (anger, fear, disappointment, and sadness). Presented in this section are illustrated accounts^4^ of sadness and, due to its overwhelming prevalence, the category ‘avoidance’; the illustrated accounts of fear and anger will be presented in the following section on gendered pattern of responses.

##### Sadness

Women and men both expressed sadness when imaging DOPE policies, albeit for different reasons. For example, some were sad about the fact that the policies implied restrictions in their minds:

“Well, I say to myself that (.) we live in a very sad world where, in the end, we have to (.) We have to ban.^5^ And then we'll end up prohibiting banning.” (women, 52 yo)

Others expressed sadness about lost potential linked to the actual policy topic, in this case the loss of being able to travel freely:

“(.) Er, I'm a bit sad. (h) […] To not be able to travel anymore, because I haven't been out of Europe too much so far, because of lack of money and I'm looking forward to going to other countries and if I couldn't do it anymore, well yeah for sure, I'd be really sad, yeah.” (women, 24 yo)

##### Avoidance

The code avoidance was used to regroup five different strategies employed to avoid responding with emotional content. Namely participants (1) stated their belief about the efficacy of the policy, (2) described what would provoke an emotion but not the emotion itself, (3) explicitly avoided identifying an emotion, (4) gave an answer to a different question altogether, or (5) stated their belief about the difficulty to implement the policy. While strategies 3 and 4 will be illustrated in the next section, strategy 5 (illustrated here) shows how instead of verbalising an emotion, the participant exposes his projection about the difficulty of finding balance between expected results and seemingly difficult to accept policies.

“(…) Well:: indeed, for people who want children, I understand that it's very complicated to accept things like that. (interviewer: uh hun). and if you want to have a: result at the end, you have to apply the measures well. But that's what I find difficult, how to find the right middle ground? (man, 49 yo)”

**Figure 2:**
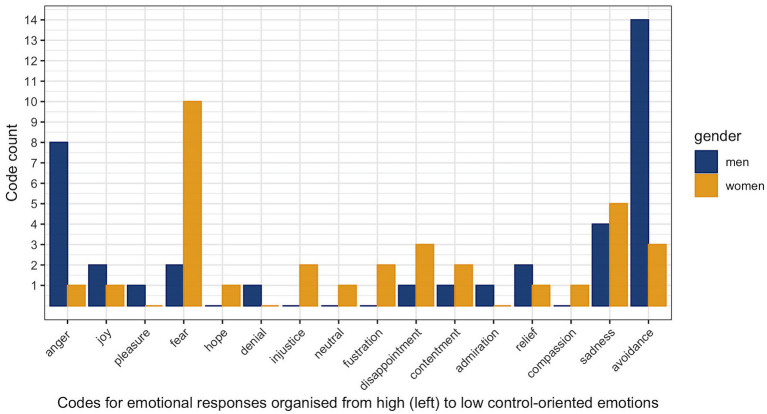
Count for each emotion code by gender.

As can be seen in Figure 1, over the 21 interviews conducted there were 18 coded instances of avoidance. This outlines that the most common emotional response was one with no emotional content at all. Although it is an interesting finding, the whole story is told when splitting the data by gender (as can be seen in Figure 2).

#### Gendered Pattern of Emotional Responses—Asymmetry as a Function of Control Orientation

Although the prevalence of different emotions is usually quite comparable between both genders, splitting the data by gender reveals two interesting asymmetries. First, men tend to avoid emotional content more than women do, and second, women report more accounts of fear (less control-oriented) and men report more accounts of anger (more control-orientated); see Figure 2.

##### Avoidance

Let us first consider the avoidance code. We described the five strategies employed by participants to avoid expressing emotional content. Of those five strategies, two were uniquely present in male responses in our sample. The first was answering a different question; where participants glide over the question and give their opinion about a different topic, thus avoiding expressing any emotional content at all. The second, and perhaps most extreme strategy, is explicit avoidance, illustrated by the following excerpt:

“uh pff:: (.) me, I leave the emotional side of things aside a bit because, given my background uh, it's not necessarily what plays a primary role uh: (.) in the analysis of situations […] (not everyone) has an emotion about it, it's a point of view, it's uh (…) a way of seeing it isn’t it.” (man, 45 yo)

These two avoidance strategies outline the particularities of men’s gendered responses. Furthermore, gender influenced two accounts of modal emotions, fear and anger, that differ in their control orientation.

##### Fear

Fear was predominantly cited by women; and was cited in reaction to several underlying motives like the fear of what is perceived as too extreme:

“Yeah, what I'm afraid of with these measures is that we're falling into this vegan trend, for example, which (.) is unnatural for me. It's a good idea in terms of animal ethics, but I don't think it's (.), not where it should. I'd rather go back to the diet our grandparents had, and then cultivate the land that is here and then animals, yeah. Ethically.” (women, 20yo)

Other times, participants cited fear when they imagined that the policy would cause them to lose their freedom or their right to choose:

“[…] And so, I like, I like freedom. I like it, yeah. No, so no. It's, it's scary to know that there are people who would like to demand that of others.” (women, 71yo)

##### Anger

At the very end of the high control-oriented continuum there is anger. Whereas the accounts of fear were predominantly provided by women, the accounts of anger were predominantly provided by men. Furthermore, the motives that were initially linked to fear in women’s responses provoked anger in men; motives such as the feeling of losing their freedom. Another motive that was clearly identified by men as provoking anger was the notion of obligation. This was true even when the participant agreed with the environmental benefits of the policy:

“(…) so (.) anger, on the one hand because it obliges us, and we don't necessarily have the means to comply in that sense. (interviewer: uh hun, uh hun) but then, on the other hand, that it's a good thing, ecologically speaking and all that, it's still a good thing.” (man, 25yo)

Finally, anger was, in some instances, accompanied by an action; in the following excerpt, the participant adds the fact that he would not comply to the policy and be ‘a resister’:

“I'd be more like, uh, if you compare it to then, I'd be a resister.” (man, 25yo)

In effect, the different motives for fear, expressed predominantly by women, provoked expressions of anger in men to counter a perceived loss of freedom or perceived obligations.

### Discussion

This study explored two research questions: When faced with degrowth-oriented pro-environmental (DOPE) policies proposed by a pro-environmental activist minority, would participants respond with high control-oriented emotions? and, would men do so even more than women?

#### General Pattern of Emotional Responses

The most prevalent emotions expressed by our sample were of negative valence. This is in line with past research demonstrating that the use of degrowth oriented policies tend to create reactance and negative emotions in participants (Drews and Reese, 2018). The prevalence of negative emotions is also in line with the prediction of the biopsychosocial model of challenge and threat (Blascovich, 2014) that posits that participants who appraise a situation as a threat will react with more negative than positive emotions, as illustrated by the accounts of sadness.

Accounts of sadness were quite prevalent (for both women and men). This was unexpected, as sadness is considered a low control-oriented emotion (Sacharin et al., 2012), and is thus a negative example of our conjecture that DOPE policies would be met with high control-oriented emotions. That said, sadness is often considered as being linked to loss, either in terms of signalling loss of some kind (Leventhal, 2008) or in terms of eliciting help to avoid it (Hackenbracht and Tamir, 2010). This would be consistent with the idea that DOPE policies are perceived as a threat albeit not with the predicted control-oriented response.

Participant’s general emotional avoidance can also be understood as a way of coping with threat (unpleasant situations). For instance, the avoidance in this study is similar to the strategies that participants used to deal with unpleasant emotions related to climate change in Norway (Norgaard, 2006). Namely, participants tend to focus on what can (or cannot) be done rather than the provoked emotions. In the same vein, recent studies have shown how participants tend to use rationalisation and denial as a defence against the injunction to act pro-environmentally (Wullenkord and Reese, 2021). Referring to our conjecture that losing control would be considered a threat, participants made their discomfort with perceived loss of freedom (or loss of choice) explicit, thus highlighting the association between losing perceived control over a situation and the negative emotional outcome.

#### Gendered Pattern of Emotional Responses

As shown in the results, two aspects of the emotional responses were heavily influenced by the participants’ gender: (a) Accounts of avoidance were primarily driven by men, and (b) gender seemed to have a differential effect on emotional accounts depending on their control orientation.

##### Gendered Avoidance

Past literature makes ambiguous predictions about the expressivity of men compared to women (Kring and Gordon, 1998; Simon and Nath, 2004). Generally, though, and in line with the current findings, women in Western cultures, tend to verbally express their emotions more than men, although the same cannot be said for the physiological arousal provoked by emotional stimuli (Chaplin, 2015). This finding, however, can be linked to the notion of men being ‘internalisers’, that is, they do not display overt expressions of emotion despite physiological activation. Moreover, the lack of emotional expression found in our study could be linked to emotional suppression, a form of emotional regulation employed by participants put in powerless situations (Petkanopoulou et al., 2012). This would also be consistent with prevalent (albeit contested) western discourse that ‘boys do not cry’, predicting that men would not displaying direct emotional involvement as to not appear vulnerable (McQueen, 2017; Rafanell et al., 2017).

##### Gendered Emotional Accounts and Control Orientation

The two most prevalent emotions in our sample, however, were fear and anger. Although both can be considered as oriented towards control, anger is more control-oriented than fear (Sacharin et al., 2012). These findings are directly linked to the conjecture that DOPE policies would represent a threat that would be met with control-oriented emotions (Stephan et al., 2016). Crucially to our conjectures, though, are the findings that men and women differ in their control-oriented expressions of emotions. Women reported more fear than men and men reported more anger than women. These findings are consistent with the stereotypes of gender-consistent emotions (Brody and Hall, 2010; Brody et al., 2016). Notably, of all the emotions that have been highlighted as being more often expressed by women (Fischer et al., 2004; Brody et al., 2016) fear is the most control-oriented (Scherer et al., 2013a), often aimed at avoiding uncertainty (Lerner and Keltner, 2000; Nabi, 2003). For men, however, not only is anger the most stereotypical self-expressed emotion (Brody et al., 2016; Gansen and Martin, 2018) but is also considered the most control-oriented emotion overall (Scherer et al., 2013a). Crucially, these findings echo with previous findings of how men display anger because they feel they have the ability to change the outcome of a situation (Lerner and Keltner, 2000), for instance, by using anger to maintain their social power (Timmers et al., 1998; Fischer et al., 2004; Schwalbe, 2015).

#### Interim Conclusion

Put together, these findings provide tentative evidence towards the study’s conjecture that DOPE policies proposed by an active minority would be perceived as a threat and that the control-oriented responses they elicit would be gendered, with men expressing more high control-oriented emotions (here, anger) than women. The following sections describe studies devised as a quantitative test of the findings provided by this qualitative analysis. The derived hypotheses were that, when faced with DOPE policies proposed by an active minority, participants will select more high than low control-oriented emotions on the Geneva Emotion Wheel, and that men will do so more than women.

## Pilot Study

To test the above hypotheses, we exposed participants to the same minority message (bogus manifesto extract) used in study 1, and then asked them to identify their emotional responses on the GEW.

### Method

#### Participants

Participants were psychology undergraduates attending a Swiss university (*M_age_ =* 21.7; *SD* = 3.42); they participated for course credits. The final convenience sample was comprised of 138 students, 107 of which were women.^6^

#### Procedure

The procedure was comparable to that in study 1, only adapted to an online design where, instead of interviews, participants reported their emotions using the GEW. First, participants were welcomed by a page stating that the study was interested in their emotions linked to climate change, before being asked to complete an ethics form. Then, participants were told that, as the study was interested in perceived personal consequences related to climate change, and as the research team was unsure about the different information participants have, they would be asked to read the manifesto extract supposedly written by a pro-environmental activist group, ‘Alarm Swarm’ about what everyone should do to counteract the climate crisis (same as study 1). Once read, participants were led to the next survey page where they were presented with the first environmental problem and related DOPE policy, for which they were asked to select what emotion(s) the policy made them feel. This process was repeated for all three policies before participants were led to the final page containing demographic questions (gender, age, nationality, highest degree received, and working status). The final message thanked them for their participation, and just like in study 1, explained the gist of the study and invited them to send any questions to the research team if needed.

#### Materials

The study was designed as an online survey using Qualtrics (Qualtrics, 2019, https://www.qualtrics.com).

##### Geneva Emotion Wheel

Participants emotional responses to each policy were measured using the French version of the Geneva Emotion Wheel (GEW; Scherer et al., 2013a,b).^7^ The tool was created and validated in a Swiss context very similar to the one in which the study was conducted. The GEW presents 20 emotions in a circle, organised on two orthogonal axes: positive to negative valence (right to left) and high (top) to low (bottom) control-oriented emotions, allowing us to use a theoretically driven measure of control-oriented emotions.

##### Dependent Variables

Two different dependent variables were used to test our hypotheses:

1. The *overall frequency* (that is, summed across all three policies) of control-oriented emotions chosen by men and women.
2. The *proportion of high control-oriented emotions*. Each participant received a score calculated by the total amount (summed across all policies) of high control-oriented emotions they selected, divided by the total amount of emotions they selected. A proportional score is particularly relevant, as it allows to circumvent the higher number of emotions reported by women as compared with men. Furthermore, the data was weighted as a function of each emotion’s position on the control axis, in the following manner. We ascribed relative weightings to each emotion depending on its position within the GEW. The emotions that were at the very top and bottom position (i.e., very high- and very low-control emotions: anger, interest, sadness, and compassion) received a weighting of 5. The weightings decreased incrementally leaving the last four emotions (those on both sides of the middle of the control-axis) with a weighting of 1.

### Results

#### Overall Frequencies

When faced with degrowth-oriented pro-environmental policies, expectedly although non significantly, there was a greater count of participants selecting more high control-oriented emotions than low control-oriented emotions (67) than participants selecting more low control-oriented emotions than high control-oriented emotions (49) on the Geneva Emotion Wheel, χ^2^(1, 116) = 2.79, *p* = 0.09 Cohen’s *w* = 0.16. Furthermore, the gender differences were in the expected direction, with men selecting more high than low control-oriented emotions to a higher extent than women, although the difference was not significant, Chi-Square Goodness of Fit Test, χ^2^(1, 116) = 0.05, *p* = 0.82, Cohen’s *w* = 0.04 (see Supplementary Table S2).

#### Proportions

When observing the weighted data^8^ for the proportional-dependent variable, it appeared that participants did indeed select a higher personal proportion of high control-oriented emotions (*M* = 0.56, *SD* = 0.193), with the test against equal proportion level (0.50) being significant, *t*(137) = 3.59, *p* < 0.001, Cohen’s *d* = 0.31.^9^

Overall, men (*M* = 0.57, *SD* = 0.21) descriptively selected a higher individual proportion of high control-oriented emotions than women (*M* = 0.56, *SD* = 0.19), but the difference was not significant, *t*(136) = 0.35, *p* = 0.73, Cohen’s *d* = 0.069 (as can be seen in Supplementary Figure S1).

### Discussion

The pilot study aimed to test the hypotheses that when faced with a minority message advocating for degrowth-oriented pro-environmental (DOPE) policies, participants would tend to select more high than low control-oriented emotions, and that men would do so more than women. Overall, the results seemed to match the predicted pattern, but the results were not significant. The only notable exception was that participants did tend to select a greater proportion of high control-oriented emotions. However, considering the following shortcomings of the current study, the results seemed promising.

#### Study Design Limitations

Although participants’ responses were pooled for all three DOPE policies, the order in which they were presented was fixed. This could have had a spillover effect on participants’ ratings of the first onto the two subsequent policies. As discussed in Brody et al. (2016), self-reports of emotions are particularly vulnerable to participants’ situational knowledge. It is possible that once participants had given their first set of emotional responses, their desirability would influence answers in the two other policies.

Due to the employed convenience sample, only 26 men (compared to 90 women) were retained in the final Chi-square analysis; this yielded a power of 0.07, too small to detect any kind of significant effect. Moreover, the well-known problems of WEIRD sampling notwithstanding (Henrich et al., 2010), the student-based sample could have specifically biassed our study’s results. First, because the sampled age group (*M*_age_ = 21.7 years old) does not represent the majority of those confronted to DOPE policies. Second, because Western students are specifically, both in terms of their age and their social environment (university setting), a social group with high environmental attitudes and norms (Fernández-Manzanal et al., 2007; Dunlap, 2008). Third, because younger generations are less inclined towards social dominance orientation and score higher with universalism values, they would find DOPE policies less challenging than members of older generations that are more attached to the current dominant social paradigm (Duriez and Van Hiel, 2002). Fourth and finally, because of their young age, it is possible that young men’s internalised societal status is not as high as it is for older men and that, therefore, men in the students sample will not have reacted with particularly higher levels or threat response (Roberts, 2013, 2014).

In brief, study 2 suffered from severe sampling weaknesses that could have affected the results on a theoretical perspective as much as on a theoretical one. It was reported for the sake of full disclosure, and to pave the way to the design of the following study. These weaknesses were therefore addressed in study 2.

## Study 2

The aim of study 2 is to test the conjectures derived from the qualitative analyses. That is, (a) when faced with DOPE policies proposed by an active minority, participants will select more high than low control-oriented emotions on the Geneva Emotion Wheel, and (b) men will do so more than women.

### Method

#### Participants

Both sample size and participants’ demographics were addressed in study 2’s sample. Two methods for recruiting participants were used. First, an online social-media recruitment aimed at a French speaking online population that were over 35 years old; an advertisement was posted on one of the most popular online social media, with specific requirements for age group. Second, a sample was recruited from Prolific (https://www.prolific.co; Prolific, 2014; Palan and Schitter, 2018). In total 440 participants were recruited and provided full responses which should have provided a power of 0.8 for a Cohen’s *w* of 0.14 (small effect size) on the chi-square goodness-of-fit test.

The final sample (described in Table 2) was comprised of 390 participants of which *N* = 204 were women (96 from social-media) and *N* = 186 were men (118 from social-media), the power of the *t-*test was 0.88 for the weighted data. Fifty participants were dropped because they failed the attention check. The average age across both samples was *M_age_* = 43.5 years old (*SD* = 12.52, Min. = 20, Max. = 84). Although a majority had obtained a university degree, study 3’s participants declared that they were now working (73.84%) and the sample was comprised of 52% of women.

**Table 2:** Demographic information for social-media and Prolific sample.

Demographic characteristics	By sample type	
	Prolific (*n* = 214)	Social-media (*n* = 176)
Gender		
Male	118	68
Female	96	108
Age		
Mean (*SD*)	37.59 (10.91)	48.73 (11.42)
Have children %		
yes	66.74	38.71
Employment status %		
Training	2.31	6.45
Working	71.76	76.88
Both	7.87	6.99
Retired	12.04	3.76
Unemployed	6.02	5.91
Diploma (highest achieved) %		
None	1.1	1.39
Obligatory school	4.8	0.93
Vocational training	2.2	17.13
Higher education	5.9	3.70
Federal maturity	16	16.20
University degree	70	60.65

#### Procedure

The procedure was identical to the pilot study.

#### Materials

All materials, dependent variables and data weighting used in study 2 were identical to the pilot with the exception of the following three modifications made to the online survey: (1) Control variables were added (do participants have children and main country of residence) to the demographic section, (2) an attention check was added at the end of the manifesto extract page, and (3) the order of the policies was randomised and coded for to check for potential spillover effects.

### Results

#### Equivalence Test

The two sub-samples (social media vs. Prolific) were compared considering the following demographics: age, gender distribution, highest achieved diploma, and current employment and whether participants have children. Both samples were comparable across all variables apart from whether participants had children or not.^10^ However, as the control variables tests (presented in Supplementary Material G) did not affect the results, all subsequent analyses were conducted using the aggregated data.

#### Overall Frequencies

The overall frequency of participants who selected more high than low control-oriented emotions was not significantly greater than the frequency of participants who selected more low than high control-oriented emotions, due to the asymmetry across genders. Indeed, and as hypothesised, more men (than women) selected more high than low control-oriented emotions, Chi-Square Goodness of Fit Test, χ^2^(1, 328) = 3.95, *p* = 0.046, Cohen’s *w* = 0.12 (Table 3).

**Table 3:** Frequency table of men and women who selected more high than low control-oriented emotions vs. more low than high control-oriented emotions – Raw data.

Which control orientation was predominant	Men	Women	Total
More high than low control-oriented emotions	89^a^	75	164
	79.5^b^	84.5	164
More low than high control-oriented emotions	70	94	164
	79.5	84.5	164
Total	159	169	328

For each cell:

a Is the observed count.

For each cell:

b Is the expected count.

#### Proportions

**Figure 3:**
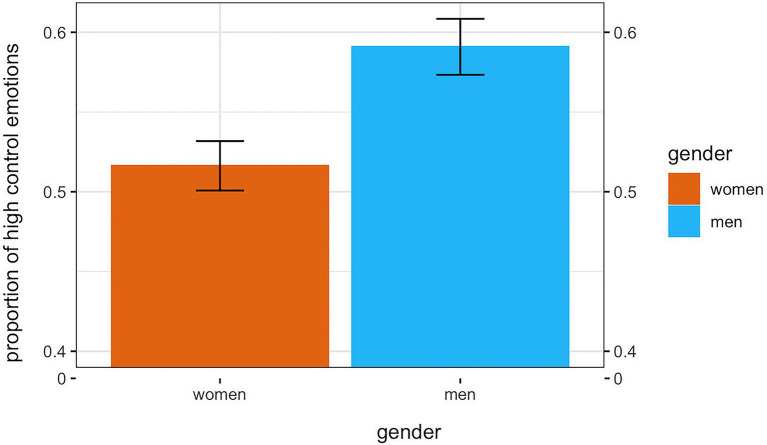
Mean gender difference in proportion of high control-oriented emotions (study 2).

As predicted, participants selected a higher proportion of high control-oriented emotions overall, as for the weighted^11^ data analysis (*M* = 0.55, *SD* = 0.23), *t(*389) = 4.39, *p* < 0.0001, Cohen’s *d* = 0.22. Moreover, men (*M* = 0.59, *SD* = 0.23) selected a higher proportion of high control-oriented emotions than women (*M* = 0.52, *SD* = 0.23), *t*(388) = 3.20, *p* = 0.001, Cohen’s *d* = 0.32 (Figure 3).^12^

### Discussion

This study supported the hypothesis that men and women report a greater proportion of high control emotions rather than low control emotions when asked to react towards degrowth-oriented policies proposed by an active pro-environmental minority. This result appeared only with the proportional weighted score that is when the level of control expressed by each emotion was taken into account. Furthermore, men reacted on both measures with an even higher proportion of high-control emotions.

## General Discussion

Together, these two studies reveal that participants engaged in differential expression of emotions when asked to consider degrowth-oriented pro-environmental (DOPE) policies proposed by a minority source. Specifically, our research pertained to a minority source that challenges the growth tenet of the current social dominant paradigm. Consistent with the conjecture that DOPE policies would elicit a threat response, participants expressed a greater proportion of high rather than low control-oriented emotions. In study 1, when participants committed themselves to freely expressing emotions in their interviews—and did not employ avoidance strategies—they mainly reported anger and fear (high-control emotions, compared with low control emotions like sadness). In study 2, where the task was to react towards the policies by selecting emotions on the Geneva Emotion Wheel (GEW), participants reported a significantly higher proportion of high-control emotions than low-control emotions, thereby supporting our first hypothesis.

This general tendency notwithstanding, the present results also revealed that men responded with more high control-oriented emotions that women did. In study 1, men who did not avoid emotional expression elaborated on the emotion of anger in particular. Women, on the contrary, were more prone to discuss about fear—a high-control emotion, albeit less than anger. The pilot study can be considered as a preliminary quantitative study; it showed promising but inconclusive results, due to several shortcomings discussed prior. Study 2, however, brought more conclusive support to our second hypothesis, showing that men did express a significantly higher proportion of high control-oriented emotions as compared to women.

### Contributions

#### Degrowth Is Considered a Threat

Our findings connect participants’ control-oriented emotional responses to threat responses in reaction to policies based on pro-environmental degrowth-oriented messages. Indeed, past research has shown that different ways of labelling pro-environmental strategies that challenge the idea of growth (e.g., “post-growth” or “prosperity without growth”) can have an effect on participants’ emotional reactions (Drews and Reese, 2018). Although our studies’ material did not actually mention the label *‘degrowth’*, they were framed as originating from a pro-environmental activist group and proposed a control in population growth, and reduction of both consumption and destruction due to large-scale human incursions into nature. It is possible, therefore, that the elicited threat responses could be due to reactance towards the restrictive nature of the policies. Other research has demonstrated that the notion of a societal sufficiency-driven pro-environmental strategy elicits particular barriers even amongst experts (Tröger and Reese, 2021). This, again, pleads in favour of considering any change to the DSP as a perceived threat. In the present studies, and in accordance with integrated threat theory, it seems that participants coped with the threat of degrowth by expressing emotions that allow them to signal the need to recover some level of control (Stephan and Stephan, 2000).

#### Gendered Responses to Threat

Overall participants’ threat response was control-oriented and exacerbated in men’s accounts (comparatively to women’s). This contributes to our research questions in two ways: (1) Previous research has connected lower levels of SDO, which is prototypically true for women compared to men, with higher levels of environmental attitudes (Milfont et al., 2013; Stanley et al., 2021). As the DSP is linked to both poor environmental attitudes and a social hierarchy that disadvantages women (Milfont and Sibley, 2014), we could expect that women might not consider DOPE policies as particularly threatening. However, on most indicators in our studies, DOPE policies were met with a threat response by men and women alike. Hence, we could read women’s control-oriented expression of emotions as further evidence to the fact that degrowth policies are considered a threat. (2) Men are specifically threatened by degrowth. Because men stereotypically express emotions only when they are coherent with their dominant and control-oriented socialisation (Schwartz and Rubel, 2005; Brody and Hall, 2010; Schwalbe, 2015), the fact that DOPE policies did indeed elicit such expressions reveals that questioning the DSP through degrowth could be a specific trigger to men. Lending further evidence to the idea that men’s attachment to the current social (hierarchical) organisation is detrimental to pro-environmental engagement (Stanley et al., 2021).

#### Emotions in Minority Influence

As noted in the introduction, minority influence research has not systematically addressed the question of emotions elicited by their actions and messages (e.g., Maass and Clark, 1984). The present research contributes to this literature by pointing to emotional reactions to radical minority messages that may potentially hinder minority influence. Admittedly, none of our studies measured attitudinal or behavioural change in targets following the minority message. However, as discussed in the previous section, emotions that put forward control-oriented responses signal that participants considered the radical, degrowth-oriented policies proposed by the minority as a threat. All respondents, but particularly men, appeared to experience a need to recover some level of control and expressed it through their emotional response.

What the possible effects of these emotional responses might be exceeds the scope of the present research and is left to speculation. Research on minority influence has shown that minorities yield different results as a function of whether the target processes the information related to the source (the minority) or the content of the message (Mugny and Pérez, 1991). It is likely that the same goes with emotions, and that the aforementioned possible resistance may be a precursor of either psychologisation and no influence, if the minority is considered as too extreme (Papastamou, 1986), or delayed influence if the message is dissociated from its (radical) source and validated (Mugny and Pérez, 1991).

### Limitations and Future Research

Although we presented data showing that Alarm Swarm and their bogus degrowth oriented manifesto were indeed perceived as less normative than a growth orientated counterpart, we did not formally manipulate the identity of the source in the main studies, for instance by presenting DOPE policies as proposed by either a minority or a majority source of influence. Such manipulation is left to future research, to the extent that degrowth can be presented as a majority position.

These studies suffer certain limitations pertaining to the collection of emotional content. First, the data is uniquely comprised of self-reports; since participants have the time to think and regulate their emotions, it is possible that the expressed emotions are biassed by participants’ stereotypical beliefs about how they should answer (regarding their culture and gender; Brody et al., 2016). Second, the data was coded for in light of a specific appraisal model of emotions. While this allows for an analysis of responses in terms of control-orientation, other theoretical frameworks should be considered, as reliance on a single model might have obstructed a different interpretation of the reported emotions (Azungah, 2018).

One other limitation of the current study pertains to the target audience of degrowth-oriented influence. Although the findings provided by studies 2 and 3 would tentatively suggest that there is a difference between student and older professional samples in terms of emotional reactions towards DOPE policies, the hypothesis predicting a formal difference between age groups would need formal testing. This is especially necessary when studying DOPE policies in light of current research on the implication of younger generations in the transition between the current dominant social paradigm and the ‘new environmental paradigm’ that holds alternative beliefs in particular about future abundance of resources and human mastery of nature (Dunlap, 2008).

### Concluding Thoughts

Was Elisabeth May right in stating that “*we are ready to make the next leap in favour of a safe and in balance planet”*? Well, the current findings do not paint a particularly hopeful answer. In the context of people’s emotional responses towards DOPE policies proposed by an active minority, people (and especially men) globally responded with more high control-oriented emotions. The issue with such control-oriented responses is that they are consistent with the hypothesis that DOPE policies are perceived as a threat to the current Social Dominant Paradigm. Which, in turn, does not bode well for the environmental *leap* that Elizabeth May and environmental activism at large are fighting for.

## Data Availability Statement

The raw data supporting the conclusions of this article will be made available by the authors, upon request.

## Ethics Statement

Ethical review and approval was not required for the study on human participants in accordance with the local legislation and institutional requirements. The patients/participants provided their written informed consent to participate in this study.

## Author Contributions

RA and FB: conceptualization, methodology, and writing—review and editing. RA: data curation, formal analysis, and writing—original draft. FB: funding acquisition, project administration, and supervision. All authors contributed to the article and approved the submitted version.

## Funding

Data collection was supported by a research support grant from the Faculty of Social and Political Sciences, University of Lausanne. Open access funding was provided by the University of Lausanne.

## Conflict of Interest

The authors declare that the research was conducted in the absence of any commercial or financial relationships that could be construed as a potential conflict of interest.

## Publisher’s Note

All claims expressed in this article are solely those of the authors and do not necessarily represent those of their affiliated organizations, or those of the publisher, the editors and the reviewers. Any product that may be evaluated in this article, or claim that may be made by its manufacturer, is not guaranteed or endorsed by the publisher.

## Footnotes

^1^“Paradigm” is used throughout the article to define a set of common worldviews, beliefs and attitudes shared by a society and that give meaning to the life that is led.

^2^While a full intersectional analysis of power dynamics would lead to a more in-depth understanding, the present research represents a preliminary step and is limited to broad representations of gender power dynamics.

^3^The results of the manipulation check belong to another larger study currently written for another article.

^4^All excerpts were originally in French and were translated into English by the first author.

^5^Ban is used in the following excerpts as a translation for ‘interdire’ which, in French, can be used in the generic sense of the word ban (~ prohibit or forbid).

^6^Relevant power issues are addressed in the Discussion.

^7^The French version of the wheel is freely available and can be found at: https://www.unige.ch/cisa/gew/.

^8^All unweighted analyses for proportion variables can be found in the Supplementary Material F.

^9^Differences in degrees of freedom are due to missing values.

^10^All graphs (Supplementary Figures S2–S6) can be found in the Supplementary Online Materials.

^11^Similarly to the pilot study, all unweighted results can be found in Supplementary Material F.

^12^All tests controlling for the potential interaction effect of gender and age (also, policy presentation order, having kids or not, age and sample origin) can be found in the Supplementary Material G; none of them are reported here due to non-significance.
