# Corrigendum: Minority influence and degrowth-oriented pro-environmental conflict: when emotions betray our attachment to the social dominant paradigm

**DOI:** 10.3389/fpsyg.2023.1228184

**Published:** 2023-06-30

**Authors:** Robert A. T. Avery, Fabrizio Butera

**Affiliations:** Institute of Psychology, University of Lausanne, Lausanne, Switzerland

**Keywords:** minority influence, emotions, degrowth, change, conflict, pro-environmental action

In the published article, there was an error in Table 3 as published. In the column “Men”, row “High control-oriented emotions” the values were reported as “89, 79.00” but should be “89, 79.5”. In the column “Men”, row “Low control-oriented emotions” values were reported as “82, 92” but should be “70, 79.5”. In the column “Women”, row “High control-oriented emotions” the values were reported as “75, 85.00” but should be “75, 84.5. In the column Women, row “Low control-oriented emotions” the values were reported as “109, 99” but should be “94, 84.5”. The corrected Table 3, including the updated totals, appears below.

**Table 3:** Frequency table of men and women who selected more high than low control-oriented emotions vs. more low than high control-oriented emotions—Raw data.

**Which control orientation was predominant**	**Men**	**Women**	**Total**
More high than low control-oriented emotions	89^a^	75	164
	79.5^b^	84.5	164
More low than high control-oriented emotions	70	94	164
	79.5	84.5	164
Total	159	169	328

For each cell: ^a^ Is the observed count and ^b^ is the expected count.

In the published article, there was also an error in Supplementary Table S2 as published. In the column “Men”, row “High control-oriented emotions” the values were reported as “16, 14.47” but should be “16, 15.02”. In the column “Men”, row “Low control-oriented emotions” values were reported as “11, 12.53” but should be “10, 10.98”. In the column “Women”, row “High control-oriented emotions” the values were reported as “51, 52.53” but should be “51, 51.98”. In the column Women, row “Low control-oriented emotions” the values were reported as “47, 45.47” but should be “39, 38.02”. The corrected Supplementary Table S2, including the updated totals, appears below.

**Supplementary Table S2:** Frequency Table of Men and Women who Selected more High than Low Control-oriented Emotions vs. more Low than High Control-oriented Emotions—Raw Data.

**Which control orientation was predominant**	**Men**	**Women**	**Total**
More high than low control-oriented emotions	16^a^	51	67
	15.02^b^	51.98	67
More low than high control-oriented emotions	10	39	49
	10.98	38.02	49
Total	26	90	116

*Note*. For each cell: ^a^ Is the observed count and ^b^ is the expected count.

In the published article, there was also an error in **Pilot Study**, *Results*, Overall frequencies. The original text stated that the analyses were run on the total emotion count when, in fact, they were conducted on the total number of participants reporting higher than lower control-oriented emotions vs. lower than higher control-oriented emotions. The corrected paragraph appears below:

“When faced with degrowth-oriented pro-environmental policies, expectedly although non significantly, there was a greater count of participants selecting more high control-oriented emotions than low control-oriented emotions (67) than participants selecting more low control-oriented emotions than high control-oriented emotions (49) on the Geneva Emotion Wheel, χ^2^(1, 116) = 2.79, *p* = 0.09 Cohen's w = 0.16. Furthermore, the gender differences were in the expected direction, with men selecting more high than low control-oriented emotions to a higher extent than women, although the difference was not significant, Chi-Square Goodness of Fit Test, χ^2^(1, 116) = 0.05, *p* = 0.82, Cohen's w = 0.04 (see Supplementary Table S2).”

In the published article, there was also an error in **Pilot Study**, *Results*, Proportions, Paragraph 1. The original standard deviation for the personal high control-oriented emotions was incorrectly provided as “0.97” but should be “0.193”. The corrected paragraph appears below:

“When observing the weighted data^8^ for the proportional dependent variable, it appeared that participants did indeed select a higher personal proportion of high control-oriented emotions (*M* = 0.56, *SD* = 0.193), with the test against equal proportion level (0.50) being significant, *t*(137) = 3.59, *p* < .001, Cohen's d = 0.31^9^.”

In the published article, there was an error in **Pilot Study**, *Results*, Proportions, Paragraph 2. The original Cohen's d effect size for the difference in men's and women's individual proportion of high and low control-oriented emotions was incorrectly provided as “0.04” but should be “0.069”. The corrected paragraph appears below:

“Overall, men (*M* = 0.57, SD = 0.21) descriptively selected a higher individual proportion of high control-oriented emotions than women (*M* = 0.56, *SD* = 0.19), but the difference was not significant, *t*(136) = 0.35, *p* = 0.73, Cohen's d = 0.069 (as can be seen in Supplementary Figure S1).”

In the published article, there was an error in **Study 2**, *Results*, Overall Frequencies.

Similarly to the pilot study, the original text for study 2 stated that the analyses were run on the total emotion count when, in fact, they were conducted on the total number of participants reporting higher than lower control-oriented emotions vs. lower than higher control-oriented emotions. The corrected paragraph appears below:

“The overall frequency of participants who selected more high than low control-oriented emotions was not significantly greater than the frequency of participants who selected more low than high control-oriented emotions, due to the asymmetry across genders. Indeed, and as hypothesised, more men (than women) selected more high than low control-oriented emotions, Chi-Square Goodness of Fit Test, χ^2^(1, 328) = 3.95, *p* = 0.046, Cohen's w = 0.12 (Table 3).”

In the published article, there was an error in **Study 2**, *Results*, Proportions. The reported *t* value for the overall difference in the weighted proportion of personal high and low control-oriented emotions was incorrectly provided as “1.84” but should be “4.39”. The corrected paragraph appears below:

As predicted, participants selected a higher proportion of high control-oriented emotions overall, as for the weighted^11^ data analysis (*M* = 0.55, SD = 0.23), *t*(389) = 4.39, *p* < 0.0001, Cohen's d = 0.22. Moreover, men (*M* = 0.59, *SD* = 0.23) selected a higher proportion of high control-oriented emotions than women (*M* = 0.52, *SD* = 0.23), *t*(388) = 3.20, *p* = 0.001, Cohen's *d* = 0.32 (Figure 3).^12^

The authors apologize for these errors and state that this does not change the scientific conclusions of the article in any way. The original article has been updated.

