# Supplementary material for: Minority Influence and Degrowth-Oriented Pro-environmental Conflict: When Emotions Betray Our Attachment to the Social Dominant Paradigm
Source: Front Psychol. 2022 Jun 29;13:899933. doi: 10.3389/fpsyg.2022.899933 (PMC9277354; doi:10.3389/fpsyg.2022.899933)
Supplement: Supplementary file 1 [file Data_Sheet_1.docx]

Supplementary Online Material

Table of Contents

[1 SOM A – French Interview Script for Study 1 2](#_Toc104215619)

[Accueil. 2](#_Toc104215620)

[Phase d’Information – Influence Minoritaire. 2](#_Toc104215621)

[Phase d’Interview Semi-structuré. 3](#_Toc104215622)

[Fin et remerciement. 5](#_Toc104215623)

[2 SOM B – Manifesto Extract: Alarm Swarm, all studies 6](#_Toc104215624)

[3 SOM C – Table S1, study 1 7](#_Toc104215625)

[4 SOM D – Figure S1 and Table S2 10](#_Toc104215626)

[4.1 Figure S1 10](#_Toc104215627)

[5 SOM E - Figures S2 to S6 – Equivalence Test Graphs (Study 3) 11](#_Toc104215628)

[6 SOM F – Results for Unweighted proportion analyses 15](#_Toc104215629)

[6.1 Against .5 (Equal Proportion) 15](#_Toc104215630)

[6.1.1 Study 2 data 15](#_Toc104215631)

[6.1.2 Study 3 data 15](#_Toc104215632)

[6.1.3 T-test Between Men and Women 15](#_Toc104215633)

[6.1.4 Study 2 data 15](#_Toc104215634)

[6.1.5 Study 3 data 15](#_Toc104215635)

[7 SOM G - Regression models for control variables, study 3 16](#_Toc104215636)

[7.1 Control Variable Models 16](#_Toc104215637)

[7.1.1 Prolific (Samples) 16](#_Toc104215638)

[7.1.2 Age 16](#_Toc104215639)

[7.1.3 Policy Order Presentation 16](#_Toc104215640)

[7.1.4 Whether Participants have Kids 16](#_Toc104215641)

# SOM A – French Interview Script for Study 1

Démarrer l’enregistrement avec Interview du [date] pour le.la participant.e [initiales et numéro case]

Accueil. Bonjour Mme/M. merci de votre intérêt à participer à notre expérience portant sur le sujet des changements climatiques.

Avant de commencer, nous voudrions que vous lisiez attentivement ce formulaire de consentement et que – après avoir pris connaissance des termes et avoir posé les éventuelles questions complémentaires – vous le signiez. J’aimerais attirer votre attention sur le fait que vos données seront bien entendu traitées de manière anonyme et que vous pouvez jusqu’à l’anonymisation finale de vos données vous retirer vous-même ainsi que vos données de l’expérience. En outre, cette séance sera enregistrée afin de nous aider pour sa transcription.

- Merci bien.

Phase d’Information – Influence Minoritaire. Nous nous intéressons à comment les personnes perçoivent les conséquences personnelles causées par le changement climatique ainsi que la crise écologique. Comme nous ne sommes pas certains que tou•te•s nos participant•e•s aient, à l’heure actuelle, reçu les mêmes informations nous voudrions commencer par vous faire lire un extrait de manifeste écrit par un groupe d’activistes environnementaux au sujet du changement climatique et de ce que chacun devrait faire pour contrecarrer la crise écologique qui y est associée.

[l’expérimentateur•trice remet le texte d’influence minoritaire à la personne qui participe à l’expérience]

Le texte lu est le suivant :

Voici un extrait d’un manifeste écrit par un groupe d’activistes militant pour le climat :  « Essaim d’Alerte » :

« Les faits au sujet du dérèglement climatique sont les suivants : nous, l’humanité entière, sommes en crise; et les causes de cette crise sont les suivantes: la surpopulation humaine, des abus de consommation de ressources naturelles ainsi que la destruction de la nature par l’être humain. Nos gouvernements ont d’abord ignoré ces faits, et ceci pendant trop longtemps. Ensuite, ils ont mis en place des changements institutionnalisés mais qui n’était, en réalité, que des changements de façade.

C’est pourquoi nous devons agir, sans quoi notre avenir et l’avenir des générations futures sera sans espoir. Les conséquences imminentes de la crise sont les feux sauvages, le manque d’eau potable, la fonte des glaciers, la montée des niveaux de la mer, les pertes de biodiversité, et la liste n’est pas exhaustive ! De plus, ces pertes combinées vont amener au déplacement de millions d’individus ainsi qu'à une hausse sans égale de potentiels guerres et conflits.

Les actions humaines sont à la source de la crise écologique. C’est pourquoi, alors que nos gouvernements se complaisent à perpétuer le système mis en place, nous devons changer. Nous devons prendre notre responsabilité et agir. Afin de combattre cette crise, trois grandes causes ont été identifiées : la surpopulation humaine, l’abus de consommation de ressources naturelles ainsi que la destruction de la nature par l’être humain. Mais nous pouvons changer !

Premièrement, pensons aux générations futures et limitons les naissances futures autant que possible. Pensons à l’adoption et à l’éducation de celles et ceux qui sont déjà parmi nous !

Deuxièmement il nous faut réduire notre consommation de ressources naturelles ! Arrêtons de prendre la voiture, arrêtons de gaspiller notre eau et arrêtons d’acheter et de consommer autant de produits. Réduisons notre empreinte carbone au minimum !

Troisièmement, nous devons agir afin d’empêcher les organisations qui détruisent la nature à large échelle. Nous devons arrêter de cautionner l’élevage intensif de viande qui rase des forêts entières pour la monoculture de maïs. Une monoculture qui utilise des pesticides qui eux aussi détruisent la biodiversité. Nous devons arrêter de prendre l’avion car la pollution de l’excès de trafic aérien augmente de façon trop importante l’acidité de nos océans et détruit le monde marin.

Si nos gouvernements ne sont pas capables de nous dire la vérité sur l’état actuelle de notre planète et de mener nos actions dans la bonne direction, alors il faut que nous prenions les devants. Il ne s’agit plus de mener une vie meilleure, nous devons changer notre fonctionnement pour changer le fonctionnement de notre société. Limitons la surpopulation humaine, réduisons la consommation de ressources naturelles et arrêtons la destruction de la nature par l’être humain.

Habitant•e•s de la terre, changez le futur de notre monde : Maintenant ! »

Phase d’Interview Semi-structuré. Merci d’avoir lu le texte. Avez-vous des questions par rapport à celui-ci ? […]

Avant de vous poser la première question, je tiens à vous rappeler que suis que chercheur/euse sur ce sujet et que seule votre opinion honnête nous intéresse.

Maintenant je vais vous présenter quelques exemples de mesures proposées pour contrer la crise climatique. Vous en connaissez peut-être d’autres. Vous êtes libres de les imaginer aussi.

Très bien […] Afin de combattre la crise écologique, certaines personnes (comme vous avez pu le lire) ont suggéré qu’il y a 3 grandes causes dont la population devrait s’occuper…

(Question 1) … La première est le contrôle de la croissance de la population humaine. Par exemple, certaines mesures prise par le gouvernement en Inde (récompense financière pour les hommes prêts à faire une vasectomie) ou en Chine - la loi de l’enfant unique.

Comment percevez-vous le contrôle de la croissance de la population comme moyen d’atténuer la crise écologique (?)

*(Si la question de la perte n’est pas abordée) :*

Pensez-vous que ces mesures représentent une perte ?

En quoi pensez-vous que pensez-vous que ces mesures représentent une perte pour vous personnellement ?

Si non, pensez-vous au contraire que ces mesures représentent une opportunité ?

Lorsque vous pensez à ce type de mesures, quelles sont les émotions que vous ressentez ?

(Question 2) … Le deuxième axe d'action proposé par les militant•e•s est la réduction de consommation de ressources naturelles. Les ressources surconsommées sont les énergies fossiles (carburant, etc…), ainsi que les matières premières et l'eau qui sont gaspillées.

Par exemple, une initiative proposée par Bâle Ville (Suisse) et acceptée par le peuple demande la mise en place d'une interdiction de tous véhicules non-écologique sur leurs routes.

D'autre mesures concernant l'industrie du textile proposent une limitation de la vente (et de l'achat) à 1 kg de vêtements neufs par an et par personne dès 2022.

Comment percevez-vous les différentes mesures visant à réduire la consommation de ressources naturelles

*(Si la question de la perte n’est pas abordée) :*

Pensez-vous que ces mesures représentent une perte ?

En quoi pensez-vous que pensez-vous que ces mesures représentent une perte pour vous personnellement ?

Si non, pensez-vous au contraire que ces mesures représentent une opportunité ?

Lorsque vous pensez à ce type de mesures, quelles sont les émotions que vous ressentez ?

(Question 3) … La troisième cause de la crise écologique soulevée par les militant•e•s est la destruction industrielle et à large échelle de la nature. Ces destructions industrielles sont le fruit, par exemple, d’élevages intensifs causant la déforestation ou le résultat du transport aérien dont la pollution acidifie tous nos océans.

Pour combattre cela, certaines institutions, comme des écoles primaires à Oxford, ont banni tout repas non végétarien servi par leur cafétéria.

Dans le secteur du transport aérien, deux mesures ont été proposées en France. Il s'agit d'une interdiction de tous les vols hors-Europe non justifiés, accompagnée de l'instauration d'une loterie nationale distribuant 500'000 droits à un billet d'avion par an.

Comment percevez-vous les différentes mesures visant à éradiquer le cautionnement de la destruction industrielle de la nature (?)

*(Si la question de la perte n’est pas abordée) :*

Pensez-vous que ces mesures représentent une perte ?

En quoi pensez-vous que pensez-vous que ces mesures représentent une perte pour vous personnellement ?

Si non, pensez-vous au contraire que ces mesures représentent une opportunité ?

Lorsque vous pensez à ce type de mesures, quelles sont les émotions que vous ressentez ?

(Questions démographiques) à mettre pendant l’enregistrement ou pas ?

Genre,

Age exact

Profession ou occupation

Auto description du niveau de revenu

Fin et remerciement. Je vous remercie de votre participation et d’avoir partagé avec nous vos pensées et opinions. Avant de terminer cette session, nous aimerions savoir si vous avez encore une question ou un commentaire par rapport à cette étude. Si non, encore merci et nous vous souhaitons une très belle (fin de) journée.

# SOM B – Manifesto Extract: Alarm Swarm, all studies

**L'extrait suivant s'agit d'un extrait de pamphlet écrit par Essaim d'Alerte - groupe activiste militant pour l'environnement. Merci de bien vouloir le lire attentivement.**

Les faits au sujet du dérèglement climatique sont les suivants : nous, l’humanité entière, sommes en crise de surpopulation, d'abus de consommations de ressources naturelles ainsi que la destruction de la nature par l’être humain. Nos gouvernements ont d’abord ignoré ces faits, mais pendant bien trop longtemps. Ensuite, ils ont mis en place des changements institutionnalisés mais qui n’étaient, en réalité, que des changements de façade.

C’est pourquoi nous devons agir, sans quoi, notre avenir et l’avenir des générations futures sera sans espoir. Les conséquences imminentes de la crise sont les feux sauvages, le manque d’eau potable, la fonte des glaciers, la montée des niveaux de la mer, les pertes de biodiversité, des températures invivables ; la liste n’est pas exhaustive ! De plus, ces pertes combinées vont amener au déplacement de millions d’individus ainsi qu’une hausse sans égale de potentielles guerres et conflits.

Les actions humaines sont à la source de la crise écologique. C’est pourquoi, alors que nos gouvernements se complaisent à perpétuer le système mis en place, nous devons changer. Nous devons prendre notre responsabilité et agir. Afin de combattre cette crise, trois grandes causes ont été identifiées : la surpopulation humaine, l’abus de consommation de ressources naturelles ainsi que la destruction de la nature par l’être humain. Mais nous pouvons changer !

Premièrement, pensons aux générations futures et limitons les naissances futures autant que possible. Pensons à l’adoption et à l’éducation de celles et ceux qui sont déjà parmi-nous !

Deuxièmement, nous devons agir afin d’empêcher les organisations qui détruisent la nature à large échelle. Nous devons arrêter de cautionner l’élevage intensif de viande qui rase des forêts entières pour la monoculture de maïs. Une monoculture qui utilise des pesticides qui eux aussi détruisent la biodiversité. Nous devons arrêter de prendre l’avion car la pollution de l’excès de trafic aérien augmente de façon trop importante l’acidité de nos océans et détruit le monde marin.

Troisièmement, il nous faut réduire notre consommation de ressources naturelles ! Arrêtons de prendre la voiture, arrêtons de gaspiller notre eau et arrêtons d’acheter et de consommer autant de produits. Réduisons notre empreinte carbone au minimum.

Si nos gouvernements ne sont pas capables de nous dire la vérité sur l’état actuel de notre planète et de mener nos actions dans la bonne direction, alors il faut que nous prenions les devants. Il ne s’agit plus de mener une vie meilleure, mais de changer notre fonctionnement afin de changer le fonctionnement de notre société. Limitons la surpopulation humaine, réduisons la consommation de ressources naturelles et empêchant la destruction de la nature par l’être humain.

Habitant•e•s de la terre, changez le futur de notre monde : Maintenant !

# SOM C – Table S1, study 1

| **Table S1** |  |
| --- | --- |
| *Data Analysis Protocol (Thematic Analysis & adjustments for CQR* | |
| 6-phase approach to Thematic Analysis (Braun & Clarke, 2006) and Consensual Qualitative Research (Masdonati et al. 2017) | Work conducted  (research team members (TM): are anonymised TM1, TM2 and TM3) |
| 1 - Familiarise yourself with your data  *“Transcribing data (if necessary), reading and re-reading the data, noting down initial ideas.” (Braun & Clarke, 2006)* | Conducting the interviews (TM1, TM2).  Listening the recordings (TM1)  Transcriptions (TM1, TM2)  Working on transcriptions and formatting (TM1, TM2)  From background theories, deriving number of concepts to look out for (TM1) |
| 2 - Generating initial codes  *“Coding interesting features of the data in a systematic fashion across the entire data set, collating data relevant to each code.” (Braun & Clarke, 2006)*  *“Consensus finding through discussion. First working individually, then putting findings together and searching for consensus. (As proposed by Masdonati et al., 2017)* | Discussing basic concepts (TM1, TM2)  Independent double coding for 4 transcripts on RQDA (TM1, TM2)  Discussion and consensus for the first 4 transcripts (TM1, TM2)  First 3^rd^ party review of codes (TM3)  Repeat process for next 7 transcripts (TM1, TM2)  Second 3^rd^ party review (TM3)  Code book update (RQDA) (TM1, TM2)  Coding for the final 10 transcripts and consensual discussions (TM1, TM2)  Reviewing first 4 codes for any new codes (TM1, TM2)  Discussion and reduction of multiple coded similarities (TM1, TM2) |
| 3 - Searching for emotional themes  *“Collating codes into potential themes, gathering all data relevant to each potent” (Braun & Clarke, 2006)* | Reading through code book and taking notes (TM1)  Consensus discussion about revealed topics (TM1, TM2)  Organisation of codes into ‘code categories’ (RQDA nomenclature; TM1, TM2) |
| 4 – Reviewing themes  *“Checking if the themes work in relation to the coded extracts (Level 1) and the entire data set (Level 2), generating a thematic ‘map’ of the analysis.” (Braun & Clarke, 2006)* | Across all policies, visual plotting of participants answers based on code categories (TM1)  Summary of themes for which there is more or less evidence (TM1) |
| 5 – Defining and naming themes  *“Ongoing analysis to refine the specifics of each emotional theme, and the overall story the analysis tells, generating clear definitions and names for each theme.” (Braun & Clarke, 2006)* | Presentation of themes to third party (TM1, 3)  Consensus discussion about relevance and strength of each emotional theme (TM1, 3) linked to the appraisal theory of control/oriented emotions (Sacharin et al., 2012; Scherer, 2005).  Thoughts and discussion on relevant narrative in presenting emotional self report responses towards pro-environmental DOPE policies (TM1, TM3) |
| 6 – Producing the report | Writing up the findings in a report (TM1) |

# SOM D – Figure S1 and Table S2

| **Table S2** | |  |  |
| --- | --- | --- | --- |
| *Frequency Table of Selected Emotion’s Control Orientation for Women & Men – Raw Data* | | | |
| Which control-orientation was predominant | Men | Women | Total |
| high control-oriented emotions | 16^a^  14.47^b^ | 51  52.53 | 67  57 |
| Low control-oriented emotions | 11  12.53 | 47  45.47 | 49  58 |
| Total | 27 | 98 | 125 |
| *Note.* For each cell: ^a^ Is the observed count and ^b^ is expected^.^ | | | |

## Figure S1

*Mean gender difference in proportion of high control-oriented emotions (study 2)*





# SOM E - Figures S2 to S6 – Equivalence Test Graphs (Study 3)

**Figure S 2**

*Age by sample type*


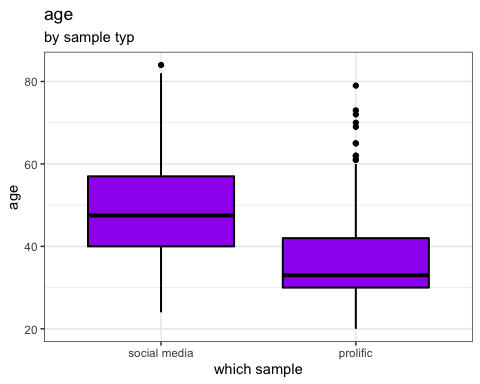


Figure S *3*

*Gender by sample type*


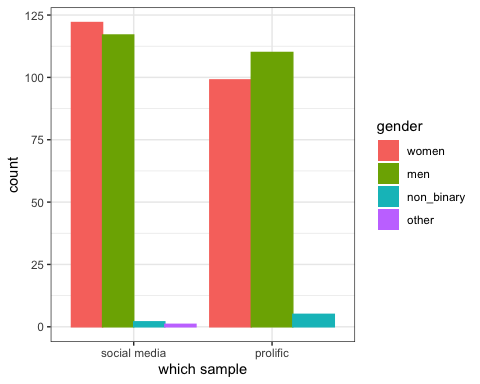


Figure S 4

*Education by sample type*


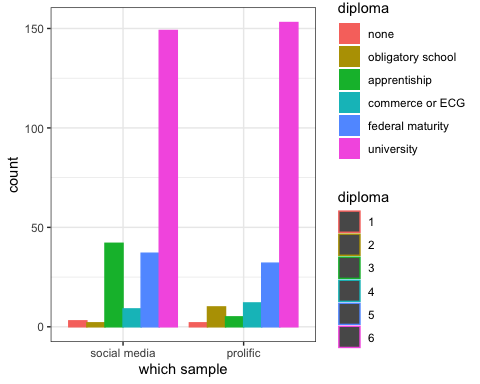


Figure S 5

*Employment by sample type*


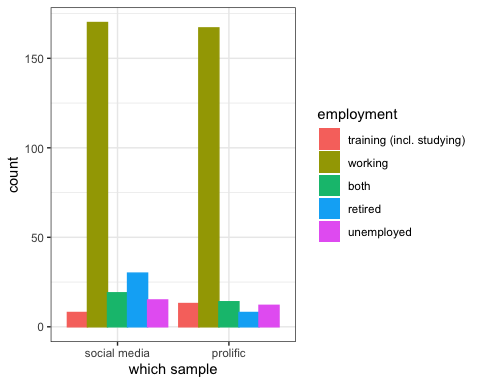


Figure S 6

*Gender by sample type*


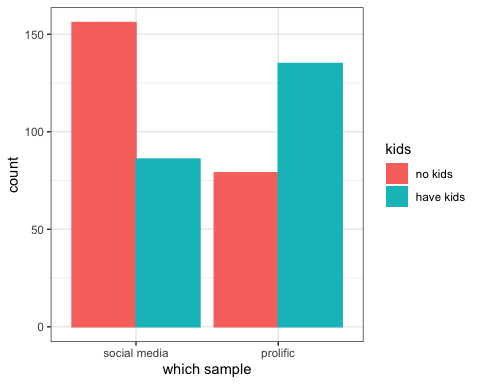


# SOM F – Results for Unweighted proportion analyses

## Against .5 (Equal Proportion*)*

### Study 2 data

Considering unweighted proportions, although participants selected a higher personal proportion of high control-oriented emotions (*M* = .53, *SD* = .17), the test against equal proportion level (.50) was only marginally significant, *t(*137) = 1.84, *p* = .07, Cohen's d = .16.

### Study 3 data

Considering unweighted proportions, although participants selected a higher personal proportion of high control-oriented emotions (*M* = .52, *SD* = .23), the test against equal proportion level (.50) was not significant, *t(*390) = 1.84, *p* = .11, Cohen's d = .08.

### T-test Between Men and Women

### Study 2 data

Overall, men (*M* = .54, *SD* = .20) descriptively selected a higher individual proportion of high (vs. low) control-oriented emotions than women (*M* = .52, *SD* = .17), but the difference was not significant, *t*(136) = 0.40, *p* = .69, Cohen’s d = .08.

### Study 3 data

Overall, men (*M* = .55, *SD* = .23) significantly selected a higher individual proportion of high (vs. low) control-oriented emotions than women (*M* = .49, *SD* = .22), *t*(388) = 0.40, *p* = .025, Cohen’s d = .23

# SOM G - Regression models for control variables, study 3

## Control Variable Models

For each control, variables as well as their interaction were added to the regression model for the weighted data. As can be observed, all effects for gender remained significant despite the added terms. We, therefore, concluded that the control variables did not affect our findings in any significant terms.

### Prolific (Samples)

(Intercept) *F*(1, 386) = 2228.47, *p* < .001, *ηp^2^*= .85 ***

gender *F(* 1, 386) = 10.16, *p* = .002, *ηp^2^*= .03 **

prolific *F* (1, 386) = 0.01, *p* = .934, *ηp^2^*< .01

gender:prolific *F* (1, 386) = 0.00, *p* = .988, *ηp^2^*< .01

### Age

(Intercept) *F* (1, 386) = 2235.50, *p* < .001, *ηp^2^*= .85 ***

gender *F*(1, 386) = 10.20, *p* = .002, *ηp^2^*= .03 **

age_1 *F* (1, 386) = 0.10, *p* = .752, *ηp^2^*< .01

gender:age_1 *F* (1, 386) = 1.13, *p* = .289, *ηp^2^*< .01

### Policy Order Presentation

(Intercept) *F*(1, 378) = 2251.68, *p* < .001, *ηp^2^*= .86 ***

gender *F*(1, 378) = 10.27, *p* = .001, *ηp^2^*= .03 **

randomization_order *F*(5, 378) = 0.71, *p* = .617, *ηp^2^*< .01

gender:randomization_order *F*(5, 378) = 1.70, *p* = .134, *ηp^2^*= .02

### Whether Participants have Kids

(Intercept) F(1, 386) = 2231.06, p < .001, *ηp^2^*= .85 ***

gender F(1, 386) = 10.18, p = .002, *ηp^2^*= .03 **

kids F(1, 386) = 0.19, p = .665, *ηp^2^*< .01

gender:kids F(1, 386) = 0.27, p = .605, *ηp^2^* < .01
